# Endoscopic submucosal dissection of a large sessile serrated lesion recurrence using an adaptive traction device

**DOI:** 10.1055/a-2078-0676

**Published:** 2023-05-10

**Authors:** Jakub Szlak, Louis-Jean Masgnaux, Jérôme Rivory, Timothée Wallenhorst, Jérémie Jacques, Michal F. Kaminski, Mathieu Pioche

**Affiliations:** 1 Department of Gastroenterological Oncology, Maria Sklodowska-Curie National Research Institute of Oncology, Warsaw, Poland; 2 Gastroenterology and Endoscopy Unit, Edouard Herriot Hospital, Hospices Civils de Lyon, Lyon, France; 3 Gastroenterology and Endoscopy Unit, Pontchaillou University Hospital, Rennes, France; 4 Gastroenterology and Endoscopy Unit, Dupuytren University Hospital, Limoges, France

Large serrated sessile lesions (SSLs) of the colon are challenging to resect completely because it is difficult to determine the margins. Therefore, recurrences are frequent and challenging to treat. For small recurrences (< 10 mm), there are a few possible resection strategies, such as underwater endoscopic mucosal resection (UEMR), endoscopic full-thickness resection (EFTR), and hot avulsion. There remains an ongoing debate as to the best way to remove larger recurrences when, because of the lesion size, EFTR risks incomplete removal (> 20 mm) or is impossible (> 30 mm) 1 . Hot avulsion usually requires several sessions to completely eradicate the recurrence. As a result, the only method for a complete single-session resection is dissection, but ESD in such lesions is challenging because of fibrosis and location (mostly in the right colon). Therefore, methods are needed to improve exposure and make dissection possible.

We present a case of ESD for a 4-cm SSL recurrence at the site of a previous piecemeal EMR performed 7 months previously in the right colon. The margins of the lesion were delineated using i-SCAN digital contrast (Pentax, Tokyo, Japan) on a therapeutic colonoscope (E34i10). An adaptive traction device (A-TRACT 2 + 2; Hospices Civils de Lyon) was used to improve the exposure of the area 2 3 4 , according to the current setup strategy 5 . After a circumferential incision with large margins (to fix the clips onto normal tissue) had been made, the first two loops were placed at the oral and anal edges of the target area ( Video 1 ), then the two additional loops were placed on the two side edges and the rubber band was fixed to the opposite wall to get 90° traction. We began the dissection process and, when exposure tended to be poor, we tightened the device to increase traction and improve exposure and visualization ( Fig. 1 ).

**Video 1** Endoscopic resection of a large sessile serrated lesion recurrence after piecemeal endoscopic mucosal resection using the adaptive traction device (A-TRACT 2 + 2).


**Fig. 1:**
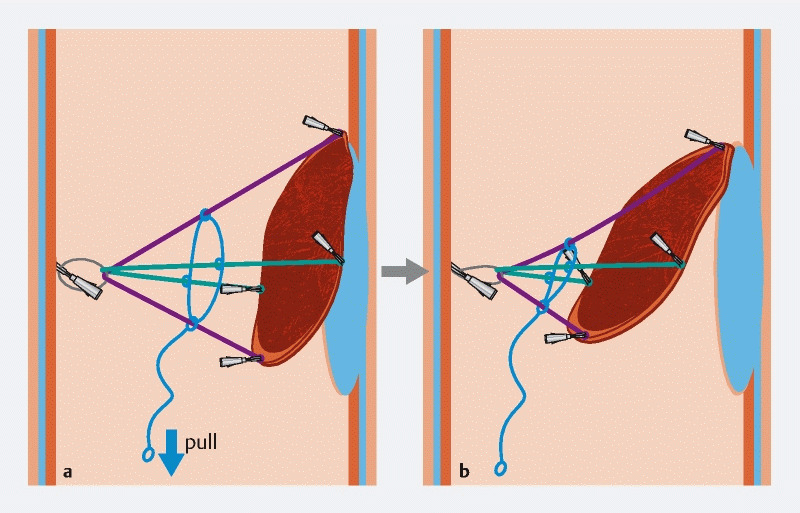
Illustration showing the adaptive traction system (A-TRACT 2 + 2) fixed on a lesion: **a** before tightening; **b** after tightening, which increases the traction during dissection.

Thanks to adaptive traction, ESD is feasible for large recurrences or difficult residual lesions, offering a chance of successful endoscopic treatment in a single session.

Endoscopy_UCTN_Code_TTT_1AQ_2AD

## Notes

Endoscopy E-Videos

https://eref.thieme.de/e-videos
*E-Videos* is an open access online section of the journal *Endoscopy* , reporting on interesting cases and new techniques in gastroenterological endoscopy. All papers include a high-quality video and are published with a Creative Commons CC-BY license. Endoscopy E-Videos qualify for HINARI discounts and waivers and eligibility is automatically checked during the submission process. We grant 100% waivers to articles whose corresponding authors are based in Group A countries and 50% waivers to those who are based in Group B countries as classified by Research4Life (see: https://www.research4life.org/access/eligibility/ ). This section has its own submission website at https://mc.manuscriptcentral.com/e-videos
